# IL-1 Receptor Antagonist (IL-1Ra) Levels and Management of Metabolic Disorders

**DOI:** 10.3390/nu14163422

**Published:** 2022-08-19

**Authors:** Kari Luotola

**Affiliations:** Faculty of Medicine, Clinicum, University of Helsinki and Helsinki University Hospital, Haartmaninkatu 4, P.O. Box 340, FIN-00029 Helsinki, Finland; kari.luotola@helsinki.fi

**Keywords:** metabolic syndrome, prediabetes, type 2 diabetes, inflammation, glucose homeostasis, diet

## Abstract

Low-grade inflammation is a major player in obesity and the metabolic syndrome predicting development of type 2 diabetes (T2DM). The interleukin-1 receptor antagonist (IL-1Ra) is a vital and natural anti-inflammatory factor and mediator in glucose homeostasis disturbances. The predictive role is independent of multiple confounders, and elevated levels appear few years before T2DM. The role of IL-1Ra is important for accumulated risk factors, dysregulated metabolism and glucose homeostasis, and dietary interventions. Longitudinal and cross-sectional population study cohorts have enabled the approximation of IL-1Ra limit values for metabolic dysregulation and guide further analysis as a potential biomarker. The limit value of IL-1Ra is reaching 400 pg/mL with prediabetes and before T2DM. However, subjects with metabolic syndrome are suggested to have lower limit values, especially among men. Future research may evaluate the role of IL-1Ra in actual glucose homeostasis together with routine fasted laboratory tests, such as glucose and C-reactive protein (CRP) instead of the oral glucose tolerance test. The significance of intermediate low IL-1Ra levels in metabolic abnormalities should be further analyzed. It is possible to specify the impact of multiple lifestyle and metabolic parameters together with age and sex. IL-1Ra could be studied in multiple approaches including interventional studies of metabolic diseases.

## 1. Introduction

Inflammation and metabolism are highly integrated where insulin resistance and beta-cell functional capacity contain common mechanisms where immune mediators have central features in altered conditions [1,2,3]. A key feature of metabolic alteration includes the integrated role of nutrients and immune responses. Quality of nutrient load and excess calories especially are involved with effects on organelle homeostasis, such as diets with increased fat or sugar [4,5,6,7]. Obesity is known as the key source of immune activation where many cytokines and other mediators increase as a hallmark for metabolic dysregulation. Inflammation is a major player in obesity, diabetes, and metabolic dysregulation, increased serum levels of low-grade inflammatory indicators exist years before the diagnosis of type 2 diabetes (T2DM), and indicators of inflammation are known to be increased [2,3]. Moreover, the level of activation is far below in situations such as acute infections. Low-grade inflammatory activity is most precisely and extensively explored in adipose tissue. The well-known indicators for subclinical inflammation include cytokine interleukin-6 (IL-6), acute phase reactant C-reactive protein (CRP), and chemokine monocyte chemoattractant protein-1.

The prevention and management of T2DM and its complications are far-reaching, even decades before clinical diagnosis. Common clinical work frequently contains situations where lifestyle or medical interventions are timely after the identification of individuals at high risk [8,9]. The presence of impaired fasting glucose or impaired glucose tolerance is an important indicator of an increased risk of T2DM. Some components of metabolic syndrome are regarded as potential guides for the assessment of intervention, but challenges remain for the applicable diagnostic tests [10,11,12]. This report evaluates the role of the specific inflammatory marker interleukin-1 receptor antagonist (IL-1Ra) as a potential clinical indicator for glucose homeostasis and its possible role in interventions for the treatment of metabolic dysregulation. Measuring IL-1Ra could direct further research and improve the specification of lifestyle, metabolic parameters, age, and sex. Improvements in the management of metabolic disorders are expected.

## 2. IL-1Ra Predicts T2DM at Population Level

The interleukin-1 (IL-1) family is an integral part of the immune system and its research has uncovered significant roles in the pathogenesis of multiple disease conditions [13]. The original IL-1 ligands are agonists IL-1 alpha (IL-1α), IL-1 beta (IL-1β), and the natural antagonist IL-1Ra; the number of IL-1 genes and family members has expanded to include agonist or antagonist ligands, receptors, and coreceptors. IL-1β plays an important role in metabolic disease and the development of diabetes that were supported in studies with a deficiency of mature cytokine or signaling leading to insulin resistance and the inhibition of IL-1β-mediated action that was shown to improve pancreatic beta-cell function [1]. Although the mechanisms leading to increased proinflammatory activation are incompletely understood, hyperglycemia has been shown to increase IL-1β expression in different cell types, including adipocytes and adipose tissue [14]. These studies direct hypotheses of hyperglycemia inducing insulin resistance through IL-1β secretion. IL-1Ra is a natural anti-inflammatory agent and inflammatory marker binding to IL-1 receptors where structural properties inhibit proinflammatory signaling and concurrently abundantly increased levels of this inflammatory marker are required to block the effects of IL-1 [15]. Furthermore, the imbalance between proinflammatory IL-1 and IL-1Ra has been suggested to predispose the development of type 1 diabetes (T1DM) and IL-1Ra was shown to block an IL-1-mediated decrease in insulin secretion in pancreatic-derived beta-cells. Altogether, the supposed counterregulatory anti-inflammatory role of IL-1Ra, protection of pancreatic beta-cell function, and altered metabolism-related proinflammatory activity are the distinct features in the development of T2DM. This makes IL-1Ra an important agent to consider as a potential marker for metabolic disorders.

Elevated blood levels of IL-1Ra can predict T2DM more than a decade before diagnosis [16]. The nested case-control analysis of the Whitehall II population-based cohort shows an independent role for IL-1Ra after adjusting for multiple confounders including waist circumference, fasting glucose, fasting insulin, CRP, and IL-6 but becomes nonsignificant with 2-hour glucose. Body mass index (BMI), waist circumference, and multiple adjustments slightly attenuated the associations. Whitehall II is a prospective cohort of civil servants and a random sample was drawn from 6058 men and 2758 women attending the phase 3 examination. Participants were followed through with postal questionnaires (2.5-year intervals) three times and clinical examinations were performed twice during the follow-up. The diagnosis of T2DM was based on (1) an oral glucose tolerance test with fasting glucose of 7.0 mmol/L or more, or a 2-hour glucose of 11.1 mmol/L or more, (2) self-reported diabetes, or (3) the use of glucose-lowering medication. The longitudinal analyses in middle-aged individuals in the Whitehall II cohort show that IL-1Ra levels increase rapidly starting at 6 years before T2DM diagnosis, independently of obesity [17]. IL-1Ra trajectories showed increased levels at baseline or at the end of the follow-up 13 years before the diagnosis (mean follow-up time was 8.8 years) with the models adjusted for age, sex, and ethnicity. The BMI- and waist circumference-adjusted models did not change the steep rise of IL-1Ra levels 6 years before a T2DM diagnosis. Subjects developing diabetes had increased age, BMI, waist circumference, blood pressure values, fasting glucose, 2-hour glucose, fasting insulin, and nonwhite ethnicity as compared with control cases.

Another study with a panel of biomarkers in middle-aged population cohorts of FINRISK97 and Health2000 shows evidence for the long-term prediction of T2DM independently of BMI, blood glucose, and other classical risk factors [18]. The FINRISK97 cohort involved subjects 25 to 75 years old in five geographical areas in Finland and was based on a representative sample from the population register with a total of 8,444 participants. The Health2000 cohort was based on a stratified two-stage cluster sampling from the population register to represent the total Finnish population aged 30 years and over with a total of 6,200 participants. Clinically incident cases during the follow-ups of 11 and 7 years were identified by a linkage with national drug registers, hospital discharge registers, and national causes for death registers. Persons with known diabetes at baseline were excluded from the analyses. IL-1Ra levels are included in these analyses with suggestive and significantly improved predictive roles for diabetes, especially among men. CRP, Apolipoprotein B100 (ApoB), adiponectin, ferritin, and IL-1Ra were the strongest predictors of T2DM among the biomarkers analyzed. IL-1Ra levels were associated with decreasing HDL-C together with increased triglycerides, ApoB, and ApoB/ApoA ratio in Health2000 and FINRISK97 cohorts.

## 3. Immune Regulation, Metabolism, and Glucose Homeostasis with IL-1Ra

The vital role of IL-1Ra as a natural anti-inflammatory factor has been demonstrated by inherited homozygous mutations of the IL-1Ra gene or large deletions at the IL-1 locus [19,20]. Increased inflammation is dependent on depleted IL-1Ra expression, and its administration rapidly resolves the symptoms. Adipose tissue is known as a major source of systemic IL-1Ra levels and is increased in conditions such as sepsis, cancer, metabolic alterations, and auto-immune diseases [21,22]. There is broad evidence for immune mediators—especially IL-1 ligands—acting in the background of major pathogenic and biological pathways for diabetes and glucose homeostasis including insulin resistance and β-cell functional capacity. The role of IL-1β versus IL-1Ra levels affecting glucose homeostasis was also evaluated. IL-1 ligands, especially IL-1β, have shown with deleterious effects on pancreatic beta-cells and IL-1Ra is protective of the insulin secretory capacity, and this anti-inflammatory action is thought to predominantly originate from adipose tissue and obesity-induced inflammation in T2DM [23]. Increased adiposity and low-grade inflammation in adipose tissue are major features of insulin resistance where IL-1 ligands are central players in proinflammation altering insulin sensitivity [24]. Obesity markedly increases IL-1Ra levels, and the levels decrease after surgery for morbid obesity [25].

Genetic studies can produce plausible evidence for the key immune factors in the background of metabolic disorders. Common genetic variation of the IL-1 family and especially single nucleotide variation of the IL-1Ra gene can explain circulating IL-1Ra levels, and the proportion of variation is modest compared to nongenetic factors [26]. The study of common gene variation in the IL-1 locus has shown a novel association of impaired glucose homeostasis with synonymous IL-1β coding single nucleotide polymorphism (SNP) and haplotype [27]. The longitudinal analyses suggested a role for IL-1 variation in the development of T2DM and the association may be gender-specific [28]. Furthermore, a large genetic analysis of seven discovery cohorts together with four replication cohorts of European ancestry revealed evidence of IL-1Ra raising by its SNP variation associated with lower fasting insulin and an improved homeostasis model assessment of insulin resistance [29].

## 4. IL-1Ra Measurements in Individuals with Metabolic Disorders

The role of IL-1Ra as a potentially useful clinical indicator is emphasized especially in situations with the presence of metabolic abnormalities such as prediabetes, impaired fasting glucose, and impaired glucose tolerance preceding T2DM [17]. Altered adiposity measurements and lipid profiles also stress disturbed metabolism, and other risk factors for T2DM and components of metabolic syndrome need to be considered. Increasing IL-1Ra is in major part explained by adiposity indicators, BMI, and waist circumference as shown in representative FINRISK97 and Health2000 cohorts [26]. Blood levels of triglycerides and total cholesterol to high-density lipoprotein cholesterol (HDL-C) ratio are among the most significant laboratory parameters associated with increasing IL-1Ra after adjusting for age and sex. A well-characterized study of 81 overweight or obese yet normoglycemic individuals reports an association of increasing ApoB and low-density lipoprotein cholesterol (LDL-C) levels with systemic IL-1Ra levels [30]. The detailed analyses including an intravenous glucose tolerance test and hyperinsulinemic euglycemic clamp showed that ApoB was associated with hyperinsulinemia, insulin resistance (IR), and IL-1Ra independently of obesity, body composition, and sex whilst the association with hyperinsulinemia and IR was dependent on IL-1Ra.

Longitudinal analysis of the FINRISK97 and Health2000 cohorts of 11 and 7 years shows that IL-1Ra levels increase at baseline with metabolic syndrome (MetS), as compared to metabolically healthy individuals and increasing IL-1Ra levels which independently predict incident T2DM after adjusting for multiple confounders such as BMI, CRP level, and even fasting glucose [28]. Among men the evidence is consistent, and women show less statistical evidence although a similar direction of association is noticed. A study of women with polycystic ovary syndrome reports increased IL-1Ra levels together with altered insulin sensitivity and increased 2-hour glucose with obesity that showed decreasing insulinogenic and glucose disposal indices associated with increasing IL-1Ra, independently of age and BMI [31]. IL-1Ra was found as a predictor for increasing 2-hour glucose independently of the BMI. Furthermore, IL-1Ra predicted an increasing 1-hour glucose at a 6-month follow-up, independently of waist circumference and CRP levels [32].

IL-1Ra measurements may be applicable for evaluating immunometabolism outcomes of nutritional interventions. A dietary intervention in a study of the healthy Nordic diet (HND) among adults with impaired glucose metabolism was followed by comparable IL-1Ra levels at baseline and after 18 to 24 weeks [33]. The randomized and controlled study was performed at six centers in Scandinavia. Inclusion criteria were age range from 30 to 65 years, BMI from 27 to 35, two criteria for metabolic syndrome (defined by International Diabetes Federation criteria), fasting glucose equal/less than 7.0 mmol/L, and a 2-hour postload glucose less than 11.0 mmol/L. The number of randomized subjects was 213. IL-1Ra levels increased within the control diet group up to a mean level of 441 pg/mL and the difference was increased (85 pg/mL; 95% confidence interval: 37–130 pg/mL) compared to the intervention group. IL-1Ra levels associated with a plasma pipecolic acid betaine (PAB) which was one of the betainized compounds analyzed and known as an indicator of fiber intake [34]. PAB levels increased together with the intervention compared to the control diet. Among other betainized compounds, only Trimethylamine N-oxide (TMAO) was slightly increased compared to the control diet. The main differences were the amounts of dietary fiber and salt, and the quality of dietary fat. Whole grain products were emphasized in the intervention group and recommended berries, fruits, vegetables, plant-based oils, margarines, low-fat dairy products, and fish whereas sugar-sweetened and dairy-fat products were limited. In the control group an intake of fish, vegetables, and bilberries was limited and the use of low-fiber cereals and dairy-fat products was emphasized. PAB and trigonelline levels were both emphasizing whole grain intake and being associated with decreased IL-1Ra, LDL-C to HDL-C ratio, and fasting insulin levels within the HND group. Increasing plasma levels of plasma pipecolic acid betaine (PAB) associated with increasing alkylresorcinols (ARs) at the end of the intervention. ARs are known as established markers of whole grain consumption. The increase in PAB with HND intervention was associated with an increased nutrient intake of fiber, magnesium, folate, and PUFA (polyunsaturated fatty acid) whereas decreased SFA (saturated fatty acid) intake was associated with a decreasing PAB. Tryptophan betaine levels showed a trend for increasing IL-1Ra at baseline and the changes of IL-1Ra and tryptophan betaine were inversely associated. Trigonelline levels were inversely associated with tryptophan betaine levels at the end of the follow-up with HND intervention.

A low-carbohydrate diet (LCD) was compared to a low-fat diet (LFD) in a study of 61 subjects with T2DM reporting a significant decrease in IL-1Ra levels in the LCD group which were also lower compared to LFD after 6 months [35]. Weight reduction and energy intake were comparable in both intervention groups and glycated hemoglobin (HbA1c) decreased significantly in the LCD group. Among other studied inflammation markers, only IL-6 levels were lower in the LCD group compared to LFD after 6 months. These results refer to an increased carbohydrate intake and altered glycemia balance on the background of high IL-1Ra levels in subjects with T2DM. This counterbalance feature is further supporting the role of endogenous or exogenous IL-1Ra as a potential therapeutic agent in diabetes.

Measuring immune activation with IL-1Ra in metabolic dysregulation among the older population is also noteworthy due to increased morbidities. A population-based cohort study of elderly individuals in a follow-up examination of the population-based Cooperative Health Research in the Region of Augsburg (KORA) shows an independent association and increased blood levels of IL-1Ra with distal sensorimotor polyneuropathy [36]. KORA was initiated to study various chronic diseases in the general population. Men and women aged 55 to 74 years were randomly selected to participate in the KORA survey and 1,209 subjects participated in the follow-up examinations. Increasing age, height, waist circumference, known diabetes, and HbA1c were associated with sensorimotor polyneuropathy in a cross-sectional setting, but total cholesterol and LDL-C levels were lower compared to the controls. Consistently, another cohort of middle-aged and elderly subjects showed increasing IL-1Ra and CRP levels associated with a presence of neuropathy among individuals with T2DM in the age- and sex-adjusted models in a large population-based Gutenberg Health Study [37].

## 5. IL-1Ra Limit Values

There are longitudinal and cross-sectional population study cohorts available for the approximation of IL-1Ra cut-off values for metabolic dysregulation and are shown in Table 1. Blood levels of IL-1Ra between 250 and 300 pg/mL could be proposed as cut-off values for increased risk of T2DM over a decade before the diagnosis and was studied in a nested case-control study within the Whitehall II cohort. However, 6 years before T2DM, the levels started to rise reaching the mean level of 399 pg/mL in the models of repeated measurements [17]. The IL-1Ra level over 408 pg/mL is proposed as a cut-off for prediabetes (defined by HbA1c between 6.0% and 6.4%) shown in Table 1 based on interquartile range (IQR) data in a large Gutenberg Health Study (GHS) [37]. GHS is a population representative single-center cohort study in western mid-Germany with a total sample of 15,010 participants. It was drawn randomly from governmental registries and stratified equally for sex, residence, and in equal strata for decades of age ranging from 35 to 74 years. The healthy Nordic diet study reported subjects with impaired glucose metabolism mean levels of IL-1Ra 366 pg/mL and 345 pg/mL at baseline [33]. Of note, females predominated with proportions of 70% and 63%. The proposed cut-off for IL-1Ra in women with MetS is between 310 and 423 pg/mL and for men between 267 and 364 pg/mL based on IQR data derived from FINRISK97 and Health2000 population cohorts [28]. MetS was defined by International Diabetes Federation (IDF) criteria.

In conclusion, the approximation of IL-1Ra limit values in metabolic dysregulation produces hints for further analysis of a potential metabolic and inflammatory marker. Several research questions remain such as what the possible role would be in evaluating actual glucose homeostasis together with routine laboratory tests. For example, fasting blood samples might produce substantial information on glucose homeostasis with IL-1Ra, glucose, and CRP when an oral glucose tolerance test would be feasible. The role of intermediate low levels of IL-1Ra in individuals with a dysregulated metabolism needs to be further evaluated. It also will be possible to specify the impact of multiple lifestyle and metabolic parameters together with age and sex with roles of increasing risk for metabolic abnormalities, T2DM, and complications. It is possible to consider IL-1Ra as an interesting marker for interventional studies of metabolic disorders.

## Figures and Tables

**Table 1:** Interleukin-1 receptor antagonist (IL-1Ra) levels in studies of population-derived cohorts.

Population Cohort Study (Ref.)		N—Case/ Non-Case	Female %	IL-1Ra Concentration (pg/mL)	IL-1Ra Concentration, Non-Case/Healthy
Whitehall II [17]	baseline T2DM cases/ non-cases	335/2475	30/27	308 (CI95%: 293–323)	248 (CI95%: 244–252)
Health2000 [28]	men with MetS/ metabolically healthy	1034/1403	0	343 (247–456)	268 (191–364)
Health2000 [28]	women with MetS/ metabolically healthy	964/1859	100	402 (272–570)	306 (213–423)
FINRISK97 [28]	men with MetS/ metabolically healthy	972/2499	0	284 (211–371)	208 (159–267)
FINRISK97 [28]	women with MetS/ metabolically healthy	666/2892	100	360 (255–458)	240 (176–310)
Gutenberg Health Study [37]	prediabetes/ normoglycemia	1425/12,152	52/50	351 (271–485)	311 (233–408)
KORA F4 [36]	elderly polyneuropathy/ no polyneuropthy	146/901	39/51	335 (248–472)	304 (233–400)

T2DM—type 2 diabetes; MetS—metabolic syndrome; IL-1Ra levels are shown as medians (interquartile range) unless indicated and means with 95% confidence intervals (CI 95%).

## Data Availability

Supporting data of the results can be found in links: https://onlinelibrary.wiley.com/doi/10.1111/j.1365-2796.2010.02294.x, https://www.ncbi.nlm.nih.gov/pmc/articles/PMC3816905/, https://diabetesjournals.org/care/article/38/7/1356/31028/Profile-of-the-Immune-and-Inflammatory-Response-in, https://www.ncbi.nlm.nih.gov/pmc/articles/PMC2857902/.

## References

[1] Hotamisligil G.S. (2017). Inflammation, metaflammation and immunometabolic disorders. Nature.

[2] Kolb H., Mandrup-Poulsen T. (2005). An immune origin of type 2 diabetes?. Diabetologia.

[3] Pickup J.C. (2004). Inflammation and activated innate immunity in the pathogenesis of type 2 diabetes. Diabetes Care.

[4] Wellen K.E., Hotamisigil G.S. (2005). Inflammation, stress and diabetes. J. Clin. Investig..

[5] Herder C., Baumert J., Thorand B., Koenig W., de Jager W., Meisinger C., Illig T., Martin S., Kolb H. (2006). Chemokines as risk factors for type 2 diabetes: Results from the MONICA/KORA Augsburg study, 1984–2002. Diabetologia.

[6] Shoelson S.E., Lee J., Goldfine A.B. (2006). Inflammation and insulin resistance. J. Clin. Investig..

[7] Schenk S., Saberi M., Olefsky J.M. (2008). Insulin sensitivity: Modulation by nutrients and inflammation. J. Clin. Investig..

[8] Tuomilehto J., Lindstrom J., Eriksson J.G., Valle T.T., Hämäläinen H., Ilanne-Parikka P., Keinänen Kiukaanniemi S., Laakso M., Louheranta A., Rastas M. (2001). Prevention of type 2 diabetes mellitus by changes in lifestyle among subjects with impaired glucose tolerance. N. Engl. J. Med..

[9] Knowler W.C., Barrett-Connor E., Fowler S.E., Hamman R.F., Lachin J.M., Walker E.A., Nathan D.M., Diabetes Prevention Research Program Research Group (2002). Reduction in the incidence of type 2 diabetes with lifestyle intervention or metformin. N. Engl. J. Med..

[10] Florez H., Marinella G., Tempro M.G., Orchard T.J., Mather K.J., Marcovina S.M., Barrett-Connor E., Horton E., Saudek C., Pi-Sunyer X.F. (2014). Metabolic syndrome components and their response to lifestyle and metformin interventions are associated with differences in diabetes risk in persons with impaired glucose tolerance. Diabetes Obes. Metab..

[11] Herman W.H., Pan Q., Edelstein S.L., Mather K.J., Perreault L., Barrett-Connor E., Dabelea D.M., Horton E., Kahn S.E., Knowler W.C. (2017). Impact of lifestyle and metformin interventions on the risk of progression to diabetes and regression to normal glucose regulation in overweight or obese people with impaired glucose regulation. Diabetes Care.

[12] Penn L., White M., Oldroyd J., Walker M., Alberti K.G.M.M., Mathers J.C. (2009). Prevention of type 2 diabetes in adults with impaired glucose tolerance: The European Diabetes Prevention RCT in Newcastle upon Tyne, UK. BMC Public Health.

[13] Dinarello C.A. (2011). Interleukin-1 in the pathogenesis and treatment of inflammatory diseases. Blood.

[14] Koenen T.B., Stienstra R., van Tits L.J., de Graaf J., Stalenhoef A.F.H., Joosten L.A.B., Tack C.J., Netea M.G. (2011). Hyperglycemia Activates Caspase-1 and TXNIP-Mediated IL-1β Transcription in Human Adipose Tissue. Diabetes.

[15] Arend W.P. (2002). The balance between IL-1 and IL-1Ra in disease. Cytokine Growth Factor Rev..

[16] Herder C., Brunner E.J., Rathmann W., Strassburger K., Tabak A.G., Schloot N.C., Witte D.R. (2009). Elevated levels of the anti-inflammatory interleukin-1 receptor antagonist precede the onset of type 2 diabetes: The Whitehall II study. Diabetes Care.

[17] Carstensen M., Herder C., Kivimäki M., Jokela M., Roden M., Shipley M.J., Witte D.R., Brunner E.J., Tabak A.G. (2010). Accelerated increase in seruminterleukin-1 receptor antagonist starts 6 years before diagnosis of type 2 diabetes: Whitehall II prospective cohort study. Diabetes.

[18] Salomaa V., Havulinna A., Saarela O., Zeller T., Jousilahti P., Jula A., Muenzel T., Aromaa A., Evans A., Kuulasmaa K. (2010). Thirty-one novel biomarkers as predictors for clinically incident diabetes. PLoS ONE.

[19] Aksentijevich I., Masters S.L., Ferguson P.J., Dancey P., Frenkel J., van Royen-Kerkhoff A., Laxer R., Tedgård U., Cowen E.W., Pham T.-H. (2009). An Autoinflammatory Disease with Deficiency of the Interleukin-1–Receptor Antagonist. N. Engl. J. Med..

[20] Reddy S., Jia S., Geoffrey R., Lorier Suchi M., Ulrich Broeckel U., Hessner M.J., Verbsky J. (2009). An Autoinflammatory Disease Due to Homozygous Deletion of the IL1RN Locus. N. Engl. J. Med..

[21] Juge-Aubry C.E., Somm E., Giusti V., Pernin V.A., Chicheportiche R., Chantal Verdumo C., Rohner-Jeanrenaud F., Burger D., Jean-Michel Dayer J.-M., Meier C.A. (2003). Adipose tissue is a major source of interleukin-1 receptor antagonist: Upregulation in obesity and inflammation. Diabetes.

[22] Perrier S., Darakhshan F., Hajduch E. (2006). IL-1 receptor antagonist in metabolic diseases: Dr Jekyll or Mr Hyde?. FEBS Lett..

[23] Donath M.Y., Shoelson S.E. (2011). Type 2 diabetes as an inflammatory disease. Nat. Rev. Immunol..

[24] Gregor M.F., Hotamisligil G.S. (2011). Inflammatory mechanisms in obesity. Annu. Rev. Immunol..

[25] Meier C.A., Bobbioni E., Gabay C., Assimacopoulos-Jeannet F., Golay A., Dayer J.-M. (2002). IL-1 Receptor Antagonist Serum Levels Are Increased in Human Obesity: A Possible Link to the Resistance to Leptin?. J. Clin. Endocrinol. Metab..

[26] Luotola K., Pietilä A., Kinnunen M., Lanki T., Loo B.-M., Jula A., Perola M., Peters A., Zeller T., Blankenberg S. (2010). Genetic variation of the interleukin-1 family and nongenetic factors determining the interleukin-1 receptor antagonist phenotypes. Metab. Clin. Exp..

[27] Luotola K., Pääkkönen R., Alanne M., Lanki T., Moilanen L., Surakka I., Pietilä A., Mika Kähönen M., Nieminen M.S., Kesäniemi Y.A. (2009). Association of variation in the interleukin-1 gene family with diabetes and glucose homeostasis. J. Clin. Endocrinol. Metab..

[28] Luotola K., Pietilä A., Zeller T., Moilanen L., Kähönen M., Nieminen M.S., Kesäniemi Y.A., Blankenberg S., Jula A., Perola M. (2011). Associations between interleukin-1 (IL-1) gene variations or IL-1 receptor antagonist levels and the development of type 2 diabetes. J. Intern. Med..

[29] Herder C., Nuotio M.-L., Shah S., Blankenberg S., Brunner E.J., Carstensen M., Gieger C., Grallert H., Jula A., Kähönen M. (2014). Genetic Determinants of Circulating Interleukin-1 Receptor Antagonist Levels and Their Association with Glycemic Traits. Diabetes.

[30] Bissonnette S., Saint-Pierre N., Lamantia V., Cyr Y., Wassef H., Faraj M. (2015). Plasma IL-1Ra: Linking hyper apoB to risk factors for type 2 diabetes independent of obesity in humans. Nutr. Diabetes.

[31] Luotola K., Piltonen T.T., Puurunen J., Tapanainen J.S. (2016). IL-1 receptor antagonist levels are associated with glucose tolerance in polycystic ovary syndrome. Clin. Endocrinol..

[32] Luotola K., Piltonen T.T., Puurunen J., Morin-Papunen L.C., Tapanainen J.S. (2018). Testosterone is associated with insulin resistance index independently of adiposity in women with polycystic ovary syndrome. Gynecol. Endocrinol..

[33] Uusitupa M., Hermansen K., Savolainen M.J., Schwab U., Kolehmainen M., Brader L., Mortensen L., Cloetens L., Johansson-Persson A., Onning G. (2013). Effects of an isocaloric healthy Nordic diet on insulin sensitivity, lipid profile and inflammation markers in metabolic syndrome–a randomized study (SYSDIET). J. Intern. Med..

[34] Tuomainen M., Kärkkäinen O., Leppänen J., Auriola S., Lehtonen M., Savolainen M.J., Hermansen K., Risérus U., Åkesson B., Thorsdottir I. (2019). Quantitative assessment of betainized compounds and associations with dietary and metabolic biomarkers in the randomized study of the healthy Nordic diet (SYSDIET). Am. J. Clin. Nutr..

[35] Jonasson J., Guldbrand H., Lundberg A.K., Nystrom F.H. (2014). Advice to follow a low-carbohydrate diet has a favourable impact on low-grade inflammation in type 2 diabetes compared with advice to follow a low-fat diet. Ann. Med..

[36] Herder C., Bongaerts B.W.C., Rathmann W., Heier M., Kowall B., Koenig W., Thorand B., Roden M., Meisinger C., Ziegler D. (2013). Association of subclinical inflammation with polyneuropathy in the older population: KORA F4 study. Diabetes Care.

[37] Grossmann V., Schmitt V.H., Zeller T., Panova-Noeva M., Schulz A., Laubert-Reh D., Juenger C., Schnabel R.B., Abt T.G.J., Laskowski R. (2015). Profile of the Immune and Inflammatory Response in Individuals with Prediabetes and Type 2 Diabetes. Diabetes Care.

